# Sensor-regulator and RNAi based bifunctional dynamic control network for engineered microbial synthesis

**DOI:** 10.1038/s41467-018-05466-0

**Published:** 2018-08-02

**Authors:** Yaping Yang, Yuheng Lin, Jian Wang, Yifei Wu, Ruihua Zhang, Mengyin Cheng, Xiaolin Shen, Jia Wang, Zhenya Chen, Chenyi Li, Qipeng Yuan, Yajun Yan

**Affiliations:** 1 School of Chemical, Materials and Biomedical Engineering, College of Engineering, The University of Georgia, Athens, GA 30602 USA; 2 Beijing Advanced Innovation Center for Soft Matter Science and Engineering, Beijing University of Chemical Technology, 100029 Beijing, China; 3 State Key Laboratory of Chemical Resource Engineering, Beijing University of Chemical Technology, 100029 Beijing, China

## Abstract

Writing artificial logic and dynamic function into complex cellular background to achieve desired phenotypes or improved outputs calls for the development of new genetic tools as well as their innovative use. In this study, we present a sensor-regulator and RNAi-based bifunctional dynamic control network that can provide simultaneous upregulation and downregulation of cellular metabolism for engineered biosynthesis. The promoter-regulator-mediated upregulation function and its transduced downregulation function through RNAi are systematically verified and characterized. We apply this dynamic control network to regulate the phosphoenolpyruvate metabolic node in *Escherichia coli* and achieve autonomous distribution of carbon flux between its native metabolism and the engineered muconic acid biosynthetic pathway. This allows muconic acid biosynthesis to reach 1.8 g L^−1^. This study also suggests the circumstances where dynamic control approaches are likely to take effects.

## Abstract (web summary)

Engineering dynamic control can improve microbial production of target chemicals. Here, the authors design a sensor-regulator and RNAi based bifunctional dynamic control network that can simultaneously and independently turn up and down cellular metabolism for engineered muconic acid production in *E. coli*.

## Introduction

A fundamental goal of metabolic engineering is to achieve optimal productivities, titers and yields of desired compounds in the selected microbial hosts to realize economic feasibility^1^. However, it is inherently difficult to achieve such a goal, since it challenges cellular homeostasis that is stringently controlled by regulatory mechanisms operating across transcriptional^2^, translational, post-translational^3^, metabolic^4,5^, signaling^6^, and epigenetic levels^7^. The effects of genetic manipulations may be buffered by the regulations when the manipulations are not adequate^8^; while in more scenarios, when the manipulations are too aggressive or over-exerted, deleterious effects may occur due to unexpected disruption of the host’s native regulations, which leads to suboptimal production outputs^9–11^. Thus, operating biosynthetic machineries in controlled modes in highly regulated cellular environments is emerging as a new and promising strategy to improve microbial synthesis efficiency.

Applying static control (or static optimization) to metabolic engineering has been attempted and proven to be effective, which includes modulating enzyme expression level through gene knockouts, engineering promoter, ribosomal binding site (RBS), and terminator, modifying enzyme properties through protein engineering, co-localizing enzymes through protein scaffolds or compartmentalization, etc^12–15^. However, the static control is usually modulated for a specific condition. Any cellular and environmental changes may impair microbial production due to the incapability of engineered strains responding to these changes dynamically^16,17^. In contrast to static control that is susceptible to cellular and environmental changes, dynamic control is a widely adopted strategy in organisms by nature and offers added benefits, which helps adapt organismal metabolic states to the changing intracellular or environmental conditions in real time. From metabolic engineering and synthetic biology standpoints, engineering dynamic control would render the biological robustness to the hosts by maintaining the cells at the optimal production states throughout all cultivation stages, leading to higher productivities, titers and yields.

By mimicking nature, engineering dynamic control has been attempted recently for improving microbial production, which demonstrated successes and potential for broader applications. For example, an engineered promoter-regulator system sensing acetyl phosphate was able to dynamically control the carbon flux distribution and improve lycopene biosynthesis^18^. A FadR-based sensor was developed to dynamically coordinate biosynthetic modules and enhanced biodiesel production^19^. Stress-responsive promoters were also identified and engineered to dynamically alleviate accumulation of toxic intermediates for production improvement^9^. In addition, a malonyl-CoA sensor-regulator was used to construct a metabolic switch to dynamically control the biosynthesis of malonyl-CoA derived fatty acids, leading to production enhancement^20^. Most recently, a pathway-independent quorum-sensing circuit was demonstrated to regulate gene expression for production optimization by sensing cell density^21^. Compared with natural regulation systems, which can achieve both upregulation and downregulation orthogonally and simultaneously at multiple levels, the developed dynamic control approaches so far have limitations in achieving multiple control functions on broader gene targets. For instance, most engineered dynamic regulations occur only at transcriptional levels, which limits the targets that can be controlled. In addition, the dynamic control in the demonstrated applications was largely constrained to mono-function with only upregulation or downregulation being achieved separately due to limited tools and techniques being developed.

Addressing these limitations and advancing nature-inspired dynamic regulation in biological systems call for novel control strategies and understanding how to implement them in complex cellular settings. When this manuscript was under review, a study reported the regulation of cellular metabolism using optogenetic tools to improve chemical production^22^. Here, we present the design of a bifunctional dynamic control network that enables switching on target biosynthetic mechanisms and switching off competing pathways in a reciprocal and escalating fashion, so as to achieve autonomous distribution of cellular resources between growth and production. The control elements consist of sensor-regulator and RNAi. The sensor-regulator triggers gene expression by sensing a cellular metabolite, which provides the upregulation function. When this function is used to initiate antisense RNA (asRNA) synthesis for repressing different target gene expression, the opposite function (downregulation) can be realized simultaneously and independently. To validate this design, we construct and characterize a non-cognate muconic acid (MA)-mediated promoter-regulator system in *E. coli*, and built a prototype of the dynamic control network to control the expression of fluorescent proteins for testing purpose. To further examine its applicability and efficiency, we apply the dynamic control network to MA biosynthesis in a step-wise fashion, which leads to much more robust MA production. More importantly, the results of this study also shed light on the questions of whether or when dynamic control can be appropriately used to achieve desired phenotypes or improved outputs.

## Results

### Characterization of an MA promoter-regulator system

**Fig. 1:**
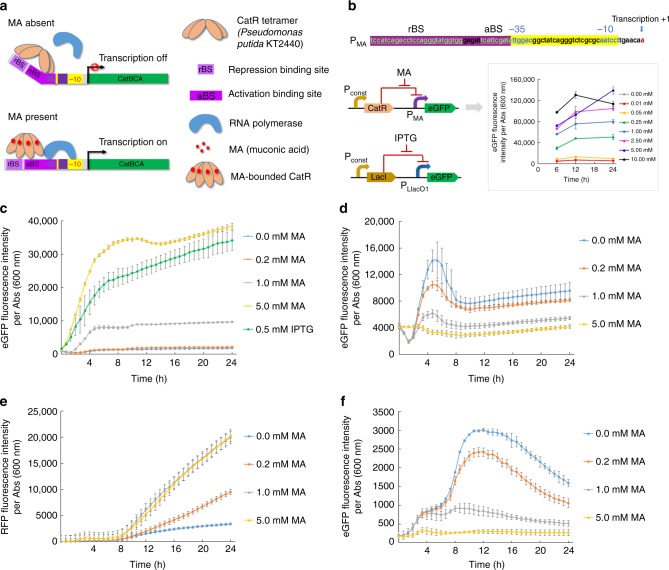
Characterization of an MA promoter-regulator system through fluorescence assays. **a** The schematic diagram of the mechanism of P_MA_-CatR system. In the absence of MA, the binding of CatR tetramer to the rBS and aBS sequences triggers a DNA bending and blocks the RNA polymerase to recognize the P_MA_, turning off the transcription of gene *catBCA*. In the presence of MA, the MA bounded CatR will trigger a conformational change, resulting in relaxing the DNA bending and recruiting the RNA polymerase to turn on the transcription of gene *catBCA*. **b** The constructs of fluorescent reporter system of P_MA_-eGFP and P_LlacO1_-eGFP and the characterization of the P_MA_ promoter with different concentrations of MA through fluorescence assays. The DNA sequence refers to the P_MA_ promoter. **c** Time-dependent gene expression dynamics of P_MA_-eGFP at different concentration (0, 0.2, 1, 5 mM) of MA. Positive control is the P_LlacO1_-controlled expression of eGFP induced by 0.5 mM IPTG, (green). **d** Dynamic downregulation of eGFP expression by RNAi under the control of the MA promoter-regulator. **e**, **f** Prototyping sensor-regulator and RNAi-based dynamic control network: **e** represents the effect of MA-responsive upregulation and **f** represents the effect of MA-responsive downregulation. The data in **b** were generated from three biological replicates. All the other data were generated from four biological replicates. The error bars represent s.d.

To construct an MA promoter-regulator system, we employed a natural MA-responsive transcription factor, CatR, from *Pseudomonas putida* KT2440. CatR is a member of the LysR family, which is a DNA-binding protein involved in regulation of the phenol and benzoate degradation gene cluster (*catBCA*) in response to MA^23–25^. The protein–protein interaction between CatR dimers forms a tetramer which binds to a 26-bp DNA sequence (termed as repression binding sequence, rBS) and a 14-bp adjacent region (termed as activation binding sequence, aBS) in the *catBCA* promoter (named P_MA_ promoter here) in the absence of MA. Since the aBS sequence overlaps with the −35 region of the *catBCA* promoter, the binding of CatR tetramer to the rBS and aBS sequences triggers a DNA bending and blocks the RNA polymerase from initiating expression of *catBCA*^24^. The binding of MA to CatR tetramer triggers a conformational change of CatR, resulting in relaxing the DNA bending and recruiting the RNA polymerase to activate the expression of downstream genes (Fig. 1a)^26^. To express CatR in *E. coli*, gene *catR* from *P. putida* KT2440 was inserted into the high-copy-number plasmid pZE12-luc under the control of a constitutive promoter P_lpp_^27^, resulting in pZE-pP_lpp_-CatR. Next, we created a reporter system by inserting P_MA_ promoter containing the 40-bp CatR binding sequence (rBS and aBS) ahead of *egfp* gene encoding an enhanced green fluorescence protein and introduced it into pZE-pP_lpp_-CatR, resulting in pZE-pP_MA_-*egfp*-pP_lpp_-CatR (Fig. 1b). In the absence of MA, CatR is expected to block the RNA polymerase by binding to the P_MA_ promoter and turn off *egfp* transcription. When MA is present, the relief of DNA bending allows the RNA polymerase to recognize P_MA_ promoter and turn on *egfp* transcription (Fig. 1b).

We evaluated the sensitivity and dynamic range of the P_MA_-CatR promoter-regulator to a broad range of MA concentrations (0, 0.01, 0.05, 0.25, 1.00, 2.50, 5.00, and 10.00 mM). As shown in Fig. 1b, when fed with MA, *E. coli* BW25113/F′ containing pZE-pP_MA_-*egfp*-pP_lpp_-CatR showed dose-dependent upregulation of fluorescence over the examined MA concentrations. More specifically, P_MA_ promoter activity was very low when MA concentrations were below 0.05 mM. We observed an obvious positive relationship between the expression strength of the P_MA_ promoter and the MA concentration in the range of 0.05–10.00 mM.

To examine the impact of changing the promoter sequence elements on the dynamic range^28^, we designed and constructed several mutated or hybrid promoters by altering the sequences between the −35 and −10 regions of the wild-type P_MA_ promoter (see Supplementary Fig. 1a). We mutated A at the −12 position of the wild-type P_MA_ promoter into T based upon the report that this mutation was able to greatly improve the promoter strength^26^, generating promoter P_mut(12AT)_. By replacing the −35 and −10 regions of the wild-type P_MA_ promoter with those of P_LlacO1_, which is a strong promoter used in *E. coli*^29^, we generated a hybrid promoter named P_hyb(−35,−10)_. In addition, another hybrid P_hyb(Llac)_ promoter was created by completely replacing the promoter sequence region of the wild-type P_MA_ promoter with the sequence spanning from the transcription start point to the −35 region in P_LlacO1_ promoter. Based upon the eGFP fluorescence assay, these mutated or hybrid promoter-regulators demonstrated varied sensitivity and dynamic range, but were not better than the wild type (see Supplementary Fig. 1b–e). Thus, we chose the wild-type P_MA_ promoter for the following study.

In addition, we speculated that the intracellular concentration of the regulator protein CatR may be another factor affecting the sensitivity and dynamic properties of the P_MA_ promoter. Higher CatR intracellular concentration may provide more stringent regulation (less leakage), but lower sensitivity. To investigate this, we amplified and inserted P_lpp_-CatR into a low-copy number plasmid pSA74 (pSA-pP_lpp_-CatR) (see Supplementary Fig. 2). Based upon the eGFP fluorescence assay, the P_MA_ promoter exhibited similar upregulation activity when CatR was either on a high-copy or low-copy number plasmid, indicating that low expression of CatR was sufficient for the stringent control of P_MA_ promoter and provided desired dynamic response. This result also suggested that P_MA_ promoter-regulator is robust to be applied in complex cellular settings.

To further investigate the upregulation function of the P_MA_-CatR promoter-regulator and the following downregulation transduced by RNAi, we conducted a series of fluorescence assays using a plate reader and representative MA concentrations (0, 0.2, 1.0 and 5.0 mM). Again, *E. coli* BW25113/F′ containing pZE-pP_MA_-*egfp*-pP_lpp_-CatR was used for the upregulation assay. As shown in Fig. 1c, a 17-fold change of eGFP expression was observed upon the addition of 5.0 mM MA at 24 h, even greater than the 15-fold change of the eGFP expression under the control of the strong P_LlacO1_ promoter on plasmid pZE-pP_LlacO1_-eGFP when induced by 0.5 mM IPTG at 24 h. These results indicated that the MA promoter-regulator system from *P. putida* KT2440 is induced by MA and even shows excellent dynamic properties in *E. coli* as a non-native cellular setting, which makes it a promising regulation tool for complex metabolic interactions.

### Dynamic downregulation by RNAi and MA promoter-regulator

RNAi is an effective technique to repress gene expression. Through introducing the asRNAs into the host system, the asRNAs are capable of regulating the expression of the target chromosomal and plasmid-carried genes by RNA–RNA interaction at the post-transcriptional level^30^. Without any modifications of the target genes, the RNAi can be flexibly implemented to control multiple targets simultaneously, especially useful for those that are essential to cell viability^31,32^. Thus, we integrated RNAi with the MA promoter-regulator to achieve downregulation function in a dynamic mode. In our previous study, we have developed asRNAs to conditionally downregulate the fatty acid biosynthesis to enhance the biosynthesis of fatty acid derived compounds^32^. The constructed asRNAs had a stem-loop structure. A pair of 38-nt inverted repeat sequences form the stem to stabilize the design; while an RNA fragment forms the loop to target a specific region of mRNA. We had demonstrated that the asRNAs with 100-nt loops binding to the mRNA translation initiation regions covering RBS and start codon provided the superior downregulation effects^32^. Thus, we kept employing the design in this study.

To enable downregulation function in response to MA, we placed the asRNAs under the control of the MA promoter-regulator system. To characterize the sensitivity and dynamic response, we also conducted eGFP fluorescence assays. First, we employed a reporter strain *E. coli* GA0 previously developed by our group, which carries a P_LlacO1_-eGFP expression cassette on the genome between *nupG* and *speC* loci^33^. Next, we designed a DNA fragment encoding a 100-nt asRNA loop sequence targeting the RBS and coding region of the *egfp* and inserted it into pZE12-luc under the control of P_MA_ promoter (pZE-pP_MA_-as*egfp*). When *E. coli* GA0 was co-transformed with pZE-pP_MA_-as*egfp* and pSA-pP_lpp_-CatR, the eGFP fluorescence intensity per absorbance at 600 nm decreased by 15, 43, and 56% within 24 h compared with the control in the absence of MA, when different concentrations of MA (0.2, 1.0, and 5.0 mM) was present (Fig. 1d). The result indicated that the MA-induced upregulation can be effectively transduced into a downregulation function through RNAi in a dose-dependent manner, which suggested that the integration of RNAi and the MA promoter-regulator could provide timely dynamic downregulation response by sensing MA concentration.

### Prototyping sensor-regulator and RNAi dynamic control network

As the next step, we aimed at introducing both the upregulation module and downregulation module into a bacterial system to examine whether simultaneous upregulation and downregulation can be achieved dynamically in an orthogonal manner, which is an important and commonly observed feature in the naturally existing dynamic regulation systems. As the prototype, we integrated the P_MA_ promoter-regulator with RNAi into a dynamic control network to regulate the expression of eGFP and red fluorescence protein (RFP) (Fig. 1e, f) and conducted fluorescence assays to characterize the sensitivity and dynamic properties of this network. As the construct of this prototype, the regulator protein CatR was constitutively expressed by the plasmid pSA-pP_lpp_-CatR, the RFP gene was placed under the control of P_MA_ promoter in plasmid pCS-pP_MA_-RFP as the reporter of the upregulation function; while the asRNA *asegfp* was also under the control of P_MA_ promoter in the plasmid pZE-pP_MA_-as*egfp* to target the *egfp* gene expression on the genome of *E. coli* strain GA0, which was used as the reporter of the downregulation function. When exposed to different concentrations (0.2, 1.0, 5.0 mM) of exogenously fed MA, the built prototype system demonstrated simultaneous and orthogonal upregulation and downregulation activities as we expected, in a dose-dependent manner. More specifically, compared with the control in the absence of MA, 2.8-, 5.9- and 6.0-fold upregulation and 34, 68, and 83% downregulation were achieved concomitantly at 24 h in the presence of 0.2, 1.0, and 5.0 mM MA (Fig. 1e, f). In general, the constructed dynamic control network in this prototype system was able to achieve the sensitivity and dynamic responses for both upregulation and downregulation functions by sensing the same inducing molecule similar to those characterized individually, although there were some differences in the time course fluorescence profiles, which is more likely due to the perturbations caused by the introduction of one additional reporter system. Overall, the results confirmed the bi-function of the designed dynamic control network at translational level.

### Comparison of dynamic and static controls

To further understand the effectiveness of the dynamic control network in regulation of metabolism for biosynthesis in engineered hosts, we first sought to anchor it to control MA biosynthesis in *E. coli* in a step-wise manner. MA is a widely-used platform chemical for biopolymers, which is commercially synthesized by petrol-derived aromatic compounds^34–36^.

**Fig. 2:**
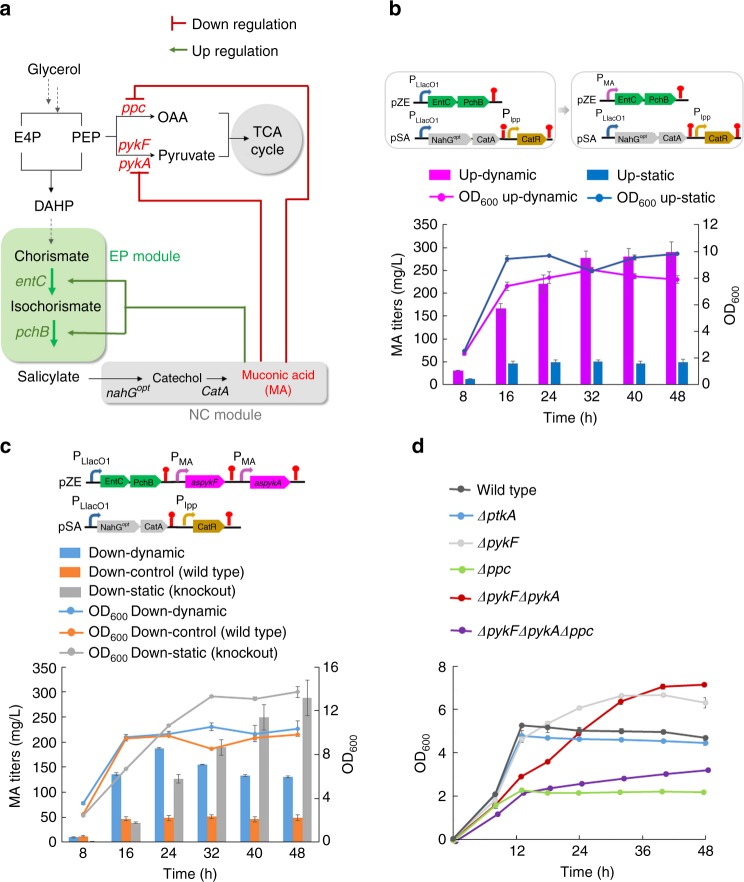
Implementation of dynamic control for MA production in wild-type *E. coli* BW25113/F′. **a** The MA biosynthesis pathway is divided into two modules: EP module (green panel) responsible for salicylate (SA) biosynthesis by EntC and PchB, and NC module (gray panel) responsible for conversion of SA to MA by NahG^opt^ and CatA. PEP flows into the tricarboxylic acid (TCA) cycle mainly through two pathways: phosphoenolpyruvate carboxylase (*ppc*)-mediated carboxylation to OAA and pyruvate kinases (*pykA* and *pykF*) mediated conversion to pyruvate. The designed dynamic control network follows the logic that the end product MA serves as the inducer to turn on the EP module and turn down the carbon flux into TCA cycle via RNAi. **b** MA production behavior by dynamically upregulating EP module. Up-dynamic: P_MA_-controlled expression of EP module (P_MA_-EP, BW25113/F′); Up-static: leaky expression of P_LlacO1_-controlled EP module (P_LlacO1_-EP, BW25113/F′). **c** MA production behavior by dynamically downregulating *pykA* and *pykF*. Down-dynamic: P_MA_-controlled expression of *aspykF and aspykA* (P_LlacO1_-EP, P_MA_-*aspykF*, P_MA_-*aspykA*, BW25113/F′); Down-control (Wild-Type): no control on gene *pykA* and *pykF* (P_LlacO1_-EP, BW25113/F′); Down-static (Knockout): *pykF* and *pykA* disruption (P_LlacO1_-EP, BW25113/F′*ΔpykFΔpykA*). **d** Growth curve of *E. coli* knockout strains with individual or combinatorial deletion of genes *pykA*, *pykF*, and *ppc*. All the above data from the static group (control) were generated from three biological replicates, all the data from dynamic group were generated from five biological replicates, and error bars represent s.d.

We previously developed a MA biosynthetic pathway that was extended from the shikimate pathway intermediate chorismate and contained two modules^37^. The upstream EP (EntC and PchB) module is responsible for salicylate (SA) biosynthesis catalyzed by isochorismate synthase (EntC) and isochorismate pyruvate lyase (PchB), and the downstream NC (NahG^opt^ and CatA) module is responsible for conversion of SA into MA catalyzed by salicylate 1-monoxygenase (NahG^opt^) and catechol 1,2-dioxygenase (CatA) (Fig. 2a). Modular optimization has demonstrated that the static control approach by expressing the EP module at a high level on pZE12-luc (pZE-EP) and expressing the NC module at a low level on pSA74 (pSA-NC) led to the maximal MA production in our previous study^37^.

With the implementation of the dynamic control network in this MA biosynthetic pathway, we expected it to have the following control logic and functions: (1) the end product MA serves as the inducing molecule; (2) in the lag phase, the cells maintain unregulated metabolism and MA pathway being turned off, allowing host cells to adapt to the fermentation environment without disturbance; (3) as cells get adapted and enter the exponential phase, the sensor-regulator and RNAi-based bifunctional dynamic control network gradually turns up the EP module and turns down the carbon flux into TCA cycle and pyruvate generation; (4) with more MA accumulation, the carbon flux is largely redirected into MA generation, realizing the transition of cellular metabolism from growth state into production state. With fulfillment of these logic and functions, the dynamic control network is expected to achieve autonomous distribution of carbon sources for growth and production in response to metabolic states by sensing MA. The initiation of these control logic and functions needs to be triggered by the low-level constitutive formation of MA as cells grow. To establish this, we constructed a two-plasmid system with EP module under control of P_LlacO1_ promoter on pZE12-luc and NC module under the control of P_LlacO1_ promoter on pSA-pP_lpp_-CatR, namely pZE-pP_LlacO1_-EP and pSA-pP_LlacO1_-NC-pP_lpp_-CatR. P_LlacO1_ promoter is an isopropyl β-d-1-thiogalactopyranoside (IPTG) inducible promoter but demonstrated leaky activity without IPTG^38^. We expected that the leaky expression of the EP module and NC module would enable the biosynthesis of MA as the initial inducing signal. To test this, we co-transferred these two plasmids into BW25113/F′ and did shake flask experiments. The generated strain produced 49.3 mg L^−1^ MA without IPTG induction in 48 h. Next, we created plasmid pZE-pP_MA_-EP to put the EP module under the control of P_MA_ promoter. When this plasmid was co-introduced into *E. coli* BW25113/F′ with pSA-pP_LlacO1_-NC-pP_lpp_-CatR, the resultant strain produced 289.5 mg L^−1^ MA at 48 h without IPTG induction. These results suggested that using the leaky expression of P_LlacO1_ promoter led to constitutive formation of MA and the MA promoter-regulator was able to sense the endogenously generated MA to provide dynamic upregulation of the upstream EP module, which improved MA biosynthesis by 5.87-fold (Fig. 2b).

As the next step, we aimed at examining the dynamic downregulation function in response to endogenously generated MA in the MA biosynthesis by employing MA-induced RNAi to redirect carbon flux into the shikimate pathway. Phosphoenolpyruvate (PEP) represents a critical metabolic and regulatory node for the biosynthesis derived from the shikimate pathway and other cellular activities such as glycolysis, TCA and growth. Starting from condensation of PEP and erythrose 4-phosphate (E4P), the shikimate pathway was restricted by the PEP availability (3% of PEP utilization) due to the existence of various competing pathways or activities, including the phosphotransferase system (PTS) sugar transport system (50%), glycolysis (15%), peptidoglycan synthesis (16%), and anaplerotic pathway (16%)^39^. During glycolysis, PEP flows into TCA cycle mainly by two pathways: (1) PykA and PykF catalyzed conversion into pyruvate that is subsequently decarboxylated into acetyl-CoA; (2) phosphoenolpyruvate carboxylase (PPC) catalyzed carboxylation into oxaloacetate (OAA) (Fig. 2a). To divert PEP into the shikimate pathway through dynamic downregulation of its competing pathways by sensing MA production, we first designed asRNAs targeting *pykA* and *pykF*, put them under the control of P_MA_ promoters respectively and introduced them into plasmid pZE-pP_LlacO1_-EP, which generated pZE-pP_LlacO1_-EP-pP_MA_-*aspykA*-pP_MA_-*aspykF* (Fig. 2c). When it was transferred into *E. coli* BW25113/F′ together with pSA-pP_LlacO1_-NC-pP_lpp_-CatR, the resultant strain produced 130.7 mg L^−1^ at 48 h without IPTG induction, which was a 2.65-fold increase compared with that (49.3 mg L^−1^) of the control strain without the MA-induced dynamic control function. It demonstrated a similar growth profile to the control strain. These results suggested that MA-induced upregulation can be effectively transduced into downregulation through the coupled RNAi at metabolic level leading to better MA synthesis and introduction of the genetic parts necessary for downregulation function did not cause any obvious stress or burden to cell viability. Next, we investigated whether the dynamic downregulation would perform better than static downregulation. Thus, we disrupted *pykA* and *pykF* genes from strain BW25113/F′, generating strain YYP167, as the control that has permanent and static downregulation of both genes. To our surprise, YYP167 carrying pZE-pP_LlacO1_-EP and pSA-pP_LlacO1_-NC-pP_lpp_-CatR produced 287.8 mg L^−1^ MA at 48 h without IPTG induction, which was around 2.20-fold higher than the titer (130.7 mg L^−1^) of the strain with dynamic downregulation (Fig. 2c). This result directly pointed out that the dynamic downregulation of *pykA* and *pykF* performed even worse than the corresponding static downregulation through disruption of *pykA* and *pykF* in MA biosynthesis. We further looked into the growth profile of the YYP167 based MA-producing strain and found that disruption of *pykA* and *pykF* generated an more biosynthesis-friendly growth phenotype, with longer growth phase and higher endpoint cell density. Based upon these results, we inferred that dynamic control of cellular metabolism is not necessarily better than the corresponding static control if the static control does not exert any negative impact onto cellular metabolism or cell viability.

### Dynamic control network improved MA biosynthesis

To further examine the above inference, we investigated the importance and impact of each of the genes *pykA, pykF*, and *ppc* on cell growth by disrupting these three genes individually and combinatorially. As the results demonstrated in Fig. 2d, when *pykA* was deleted, the knockout strain show very similar growth profile to the wild-type strain (BW25113/F′) with a slightly lower cell density during stationary phase; the strain with *pykF* knockout grew much better than both the wild-type strain and the *pykA* knockout strain with a late stationery phase; interestingly, the strain containing both *pykA* and *pykF* knockouts demonstrated even better growth featured with a longer growth phase and higher stationary cell density, which was consistent with the above growth profile during MA biosynthesis (Fig. 2c) and the previous report that *pykA* and *pykF* disruptions would be beneficial to the cell growth^40^. In contrast, disruption of *ppc* alone greatly impaired cell viability and led to significantly reduced cell growth (around 2-folds) compared to the wild-type strain. Further disrupting *pykA* and *pykF* in the *∆ppc* strain slightly restored the cell growth, which however was still significantly retarded compared with the wild-type strain. These results suggested that although *ppc* is responsible for a major PEP competing pathway, its disruption would introduce undesired burden or stress to cellular metabolism by arbitrarily limiting the carbon sources for cell growth.

**Fig. 3:**
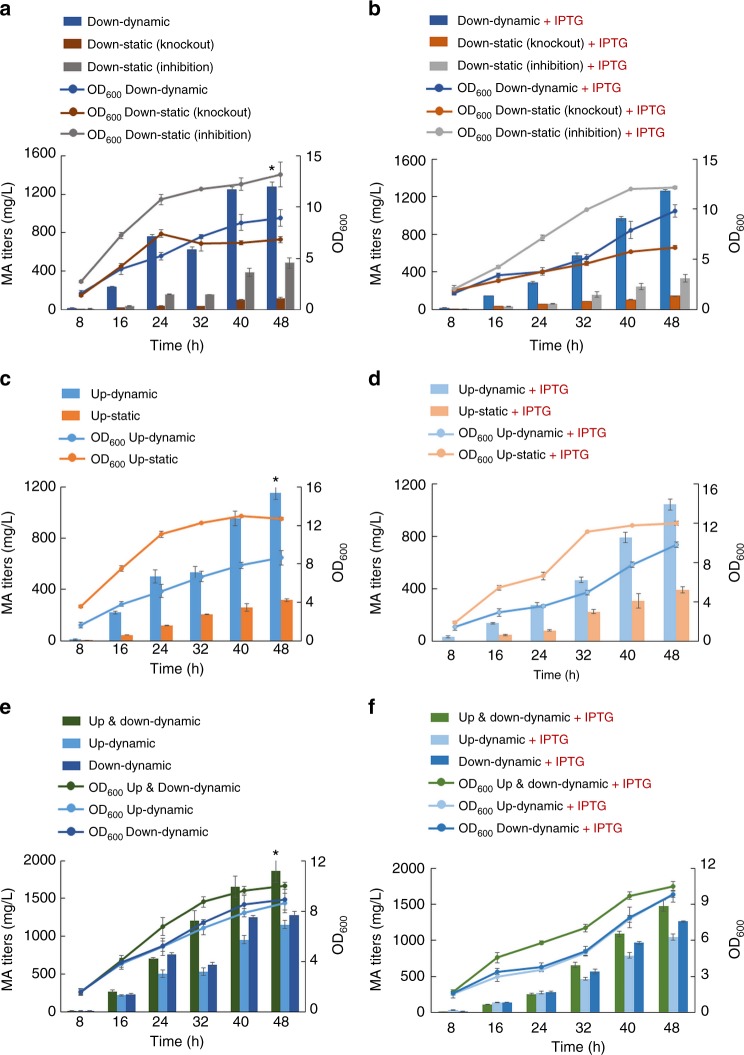
Implementation of dynamic control for MA production in *E. coli* BW25113/F′ *ΔpykFΔpykA* (YYP167). **a** MA production behavior by dynamically downregulating *ppc*. Down-dynamic: P_MA_-controlled expression of as*ppc* (P_LlacO1_-EP, P_MA_-as*ppc*, YYP167); Down-static (Knockout): *ppc* disruption (P_LlacO1_-EP, YYP167*Δppc*) and Down-static (Inhibition): leaking expression of P_LlacO1_-controlled as*ppc* (P_LlacO1_-EP, P_LlacO1_-as*ppc*, YYP167). *P < 0.05 is considered to be a statistically significant increase relative to titers from Down-dynamic in a two-tailed *t*-test. **c** MA production behavior by dynamically upregulating EP module. Up-dynamic: P_MA_-controlled expression of EP module; Up-static: leaking expression of P_LlacO1_-controlled EP module (P_LlacO1_-EP, YYP167). *P *<* 0.05 is considered to be a statistically significant increase relative to titers from Up-dynamic in a two-tailed *t*-test. e MA production behavior of dynamic upregulation and downregulation. Up & down-dynamic: P_MA_-controlled EP module and as*ppc* (P_MA_-EP, P_MA_-as*ppc*, YYP167). *P < 0.05 is considered to be a statistically significant increase relative to titers from Up & down-dynamic in a two-tailed *t*-test. The control groups are the dynamic groups of **a** and **c**. **b**, **d**, **f** Induced by 0.5 mM IPTG correspond to **a**, **c**, **e**. All the above data from the static group (control) were generated from three biological replicates, all the data from dynamic group were generated from five biological replicates, and error bars represent s.d.

Based upon these, we hypothesized that dynamic downregulation of *ppc* instead of static control through permanent disruption would render the biological robustness to host by alleviating or eliminating the metabolic stress through sensing the cellular and environment changes and then support better MA biosynthesis. To verify this hypothesis, strain YYP167 (BW25113/F′ with *∆pykA* and *∆pykF*) was employed as the host strain for MA biosynthesis and *ppc* was left as the dynamic downregulation target. When pZE-pP_LlacO1_-EP-pP_MA_-*asppc* and pSA-pP_LlacO1_-NC-pP_lpp_-CatR were co-transferred into strain YYP167, the resulting strain produced 1281.7 mg L^−1^ MA at 48 h without IPTG induction (Fig. 3a). As the controls, we also conducted MA biosynthesis in strain YYP167 with P_MA_-*asppc* replaced by P_LlacO1_-*asppc*, which provided a low-level static downregulation of *ppc* through leaky expression and in strain YYP167 with *∆ppc* (YYP171) and without carrying P_MA_-*asppc*, which provided a high-level static downregulation. As the results in Fig. 3a, these two control strains produced 483.7 and 114.1 mg L^−1^ MA, which were 2.7-fold and 11.2-fold lower than the strain with *ppc* dynamically downregulated. To further compare the robustness of these strains, we also conducted the MA biosynthesis with the presence of sufficient IPTG (0.5 mM) that enabled fully expressed MA biosynthetic pathway (EP and NC modules) in all strains and fully expressed *asppc* in the control strain containing P_LlacO1_-*asppc* that would give a moderate-level static downregulation. As the results show (Fig. 3b), the control strain with the moderate-level static downregulation produced 330.6 mg L^−1^ MA and the one with *∆ppc* produced even less (141.1 mg L^−1^), while the strain with dynamic control still produced 1264.1 mg L^−1^ MA, which represented 3.82-fold and 8.96-fold increases compared to the control strains. In addition, in both conditions (with and without IPTG) we observed that the dynamic downregulation system was able to improve the cell growth compared with the control strain containing *∆ppc* and provide a longer growth phase compared with both control strains. Overall, these results confirmed that dynamic control approaches are more likely to be effective in improving production performance or output when the corresponding static control approaches have negative impacts on cellular metabolism or cell viability.

As the next step, we examined the applicability and effect of the complete bifunctional dynamic control network in MA biosynthesis. First, we re-tested the effect of MA-mediated dynamic upregulation in the background of strain YYP167. When plasmids pZE-pP_MA_-EP and pSA-pP_LlacO1_-NC-pP_lpp_-CatR were co-transferred into strain YYP167, the resultant strain produced 1156.7 mg L^−1^ MA at 48 h without IPTG induction, which was a 3.7-fold increase compared with the control strain YYP167 (316.2 mg L^−1^) carrying pZE-pP_LlacO1_-EP and pSA-pP_LlacO1_-NC-pP_lpp_-CatR (Fig. 3c). Similarly, when both strains were induced with 0.5 mM IPTG, the EP module was fully and statically expressed in the control strain in this condition, the strain with dynamic upregulation of the EP module still performed much better than the control strain, showing a 2.65-fold increase (1043.7 vs. 394.5 mg L^−1^) in MA biosynthesis (Fig. 3d). Notably, the dynamic upregulation extended the growth phase at both conditions (with and without IPTG), although less cell density was reached. To further introduce the dynamic downregulation function, we transferred plasmids pZE-pP_MA_-EP-pP_MA_-*asppc* and pSA-pP_LlacO1_-NC-pP_lpp_-CatR into strain YYP167. Remarkably, the resultant strain produced 1861.9 mg L^−1^ MA without IPTG induction (Fig. 3e) and 1477.9 mg L^−1^ with IPTG induction (Fig. 3f). The titer was higher than those of the strains with only dynamic upregulation or downregulation as examined above, which suggested the additive effect of the orthogonal and simultaneous dynamic upregulation and downregulation. Overall, these results proved that the artificial bifunctional dynamic control network possesses enormous potential for enhanced microbial production by adapting the natural regulatory system through autonomously upregulating the desirable pathway genes and downregulating the native competing pathway genes responsive to the changing cellular physiological state.

## Discussion

Engineering dynamic control systems represents a new frontier of metabolic engineering and synthetic biology research^41^. In this study, we reported our efforts on writing artificial bifunctional dynamic control functions into native cellular regulation to reprogram cellular metabolism and improve target biosynthesis in bacteria. The bifunctional control network consists of promoter-regulator-mediated upregulation and RNAi transduced downregulation. As a proof-of-concept demonstration, a non-cognate MA sensor-regulator CatR and asRNAs were used to exemplify the design. Its functionality was first validated at translational level through fluorescence assays. Its applicability and effectiveness were further confirmed at metabolic level by efficiently reducing metabolic stresses and improving MA biosynthesis in *E. coli*. Specifically, the constructed bifunctional dynamic control network allowed MA dose-dependent upregulation of the target biosynthetic module and downregulation of the competing pathway genes encoded on genome, which significantly enhanced MA biosynthesis to 1.8 g L^−1^, substantially higher than the static controls.

During the characterization and examination of the dynamic control network, we did not spend much efforts on tuning its sensitivity and dynamic properties, since the native MA promoter-regulator and its coupling with asRNAs worked well in *E. coli*, demonstrating great robustness and desired response ranges. However, our results suggested that sensitivity and dynamic properties of the promoter-regulator component in the control network can be tuned by adjusting the binding affinity between the promoter and the regulator protein through manipulating the promoter sequence elements and regulator protein characteristics when such a need becomes necessary. Although RNAi cannot be used to realize full repression of target genes, this feature could be advantageous when it is used to downregulate the genes essential to cell viability. In addition, downregulation methods like programmable CRISPRi and protein degradation could also be anchored in the dynamic control system to achieve more diverse and sophisticated dynamic effects^42–44^. Further investigation on its applicability in regulating cellular metabolism for biosynthesis revealed that dynamic control approaches are not always superior to the static ones. In the conditions where static control approaches caused stresses to cell viability or cannot address such stresses, the corresponding dynamic control approaches may provide better solutions.

Furthermore, this work introduced an approach to regulate the PEP metabolic node to better support biosynthesis derived from PEP. Previous metabolic engineering efforts on disrupting *ppc* gene to improve PEP availability as a precursor for cellular or biosynthetic activities were not successful, due to the negative impact on cell viability^45^. However, our demonstrated dynamic strategy to regulate the PEP metabolic node was able to realize autonomous distribution of carbon flux between growth and biosynthesis at different metabolic states by sensing endogenous molecule, which can be extended to improve the biosynthesis of shikimate pathway derived products beyond the MA demonstrated here and other PEP-dependent activities^46,47^. In a broader scientific context, with many other transcription regulators or metabolite-responsive promoters that have been identified, this dynamic control network strategy can be readily applied to improve the biosynthesis of the related compounds of interest^9^. However, this dynamic control strategy still has limitations at current stage. Although various sensor-regulators responsive to a variety of metabolites were identified and characterized, there are still substantial metabolic intermediates and products in the biological systems lacking the corresponding sensor-regulators. Thus, much broader application of this control strategy still largely relies on the discovery or development of more sensor-regulators, which has been achieved at an ever-increasing rate each year. In addition, the sensor-regulator might be induced by the metabolites sharing similar chemical structures or properties with its original inducers. For example, it has been reported that the P_MA_ promoter can be induced by benzoate^23–25^. Thus, this factor should be considered when sensor-regulators are used to regulate specific pathways. Lastly, the saturation status of a sensor-regulator is an important factor worth considering when constructing a dynamic regulation network. Broad dynamic range is a desirable property for its use in metabolic engineering; however, this property may be adjusted by mutagenesis and fine-tuning of the promoter strength^21,49^.

## Methods

### Experimental materials

Luria–Bertani (LB) medium was used for *E. coli* inoculation and plasmid propagation. The modified minimal medium M9Y contains (per liter): glycerol (15 g), yeast extract (5 g), NH_4_Cl (1 g), Na_2_HPO_4_ (6 g), KH_2_PO_4_ (3 g), NaCl (0.5 g), MgSO_4_·7H_2_O (2 mmol), CaCl_2_·2H_2_O (0.1 mmol), and vitamin B1 (1.0 mg). 100 mg L^−1^ of ampicillin, 50 mg L^−1^ of kanamycin, and/or 34 mg L^−1^ of chloramphenicol were added to the medium when necessary. The *E. coli* strain XL1-Blue was used as the host strain for plasmid construction. Strain BW25113/F′ and its knockout derivatives were used for the biosynthesis of muconic acid. *E. coli* BW25113/F′ knockout derivatives BW25113/F′*ΔpykFΔpykA* (named YYP167) and BW25113/F′*ΔpykFΔpykAΔppc* (named YYP171) were created via P1 phage transduction method^48^. Plasmids pZE12-luc, pCS27 and pSA74 are respectively the high, medium and low-copy number plasmids employed for pathway assembly in this work (see Supplementary Table 1 for a list of all strains and plasmids used in this study).

P_MA_ promoter and *catR* gene (GenBank accession number SKC04124.1) were from *P. putida* KT2440 purchased from ATCC (ATCC number: 47054D-5). The eGFP gene (GenBank accession number U55762) and RFP gene (GenBank accession number AMO27245.1) were the kind gifts from Dr. Gang Cheng group at the Chemical and Biomolecular Engineering Department of University of Akron (OH, USA).

Phusion High-Fidelity DNA polymerase, restriction endonucleases and Quick Ligation Kit were purchased from New England Biolabs (Beverly, MA, USA). Zyppy™ Plasmid Miniprep Kit, Zymoclean™ Gel DNA Recovery Kit, and DNA Clean & Concentrator™-5 were purchased from Zymo Research (Irvine, CA, USA). Muconic acid standard was purchased from ACROS ORGANICS (Bridgewater, NJ, USA).

### DNA manipulation

For initial characterization of the P_MA_ promoter-regulator system, the P_MA_ promoter sequence was amplified from genomic DNA of *P. putida* KT2440 and used to replace the P_LlacO1_ promoter of pZE-pP_LlacO1_-eGFP using *Xho*I and *Eco*RI to generate pZE-pP_MA_-eGFP. To express CatR in *E. coli*, the P_lpp_ promoter sequence was amplified from genomic DNA of BW25113/F′ and gene *catR* was amplified from genomic DNA of *P. putida* KT2440. Both of them were inserted into plasmid pZE12-luc by *Xho*I, *Eco*RI, and *Xba*I, resulting in plasmid pZE-pP_lpp_-CatR. The P_lpp_-CatR operon was amplified and cloned into pZE-pP_MA_-eGFP between *Spe*I and *Sac*I, yielding pZE-pP_MA_-eGFP-pP_lpp_-CatR.

To change the dynamic response, the mutated and hybrid P_MA_ promoter sequences were synthesized and used to replace the wild-type P_MA_ promoter of pZE-pP_MA_-eGFP-pP_lpp_-CatR using *Xho*I and *Eco*RI to generate pZE-pP_mut(12AT)_-eGFP-pP_lpp_-CatR, pZE-pP_hyb(−35,−10)_-eGFP-pP_lpp_-CatR, and pZE-pP_hyb(Llac)_-eGFP-pP_lpp_-CatR. To identify the impact of CatR abundant on the P_MA_ dynamic range, the P_lpp_-CatR operon was amplified and cloned into pSA74 between *Xho*I and *Bam*HI to generate pSA-pP_lpp_-CatR.

To construct sensor-regulator and RNAi-based dynamic control network, a parent plasmid pZE-pP_MA_-PT was generated from pZE-pP_LlacO1_-PT by replacing the P_LlacO1_ promoter with P_MA_ promoter using *Xho*I and *Eco*RI. The 100 bp DNA sequence to synthesize asRNA loop targeting *egfp* was cloned into pZE-pP_MA_-PT using *Acc*651 and *Bam*HI, resulting pZE-pP_MA_-*asegfp* as asRNA synthesis plasmid. The *rfp* gene was used to replace eGFP in pZE-pP_MA_-eGFP using *Acc*651 and *Xba*I to generate pZE-pP_MA_-RFP. The P_MA_-RFP operon was subcloned and inserted into pCS27 using *Spe*I and *Sac*I, yielding pCS-pP_MA_-RFP.

To generate pZE-pP_MA_-EP, the *entC-pchB* fragment was amplified from previously constructed plasmid pZE-pP_LlacO1_-EP^37^ and was used to replace eGFP in pZE-pP_MA_-eGFP between *Acc*651 and *Xba*I. The P_lpp_-CatR operon was cloned into a previously constructed plasmid pSA-pP_LlacO1_-NC^37^ using *Spe*I and *Sac*I, yielding pSA-pP_LlacO1_-NC-pP_lpp_-CatR. The 100 bp DNA sequences to synthesize asRNA loops targeting *pykF* and *pykA* were cloned into pZE-pP_MA_-PT using *Acc*651 and *Bam*HI, resulting pZE-pP_MA_-*aspykF* and pZE-pP_MA_-*aspykA*, respectively. The P_MA_-*aspykF* and P_MA_-*aspykA* operons were cloned into plasmid pZE-pP_LlacO1_-EP using *Spe*I, *Nhe*I, and *Sac*I, yielding plasmid pZE-pP_LlacO1_-EP-pP_MA_-*aspykF*-pP_MA_-*aspykA*. Similarly, the plasmid pZE-pP_LlacO1_-*asppc* and pZE-pP_MA_-*asppc* were generated. The P_LlacO1_-*asppc* and P_MA_-*asppc* operons were cloned into pZE-pP_LlacO1_-EP or pZE-pP_MA_-EP using *Spe*I and *Sac*I, yielding pZE-pP_LlacO1_-EP-pP_LlacO1_-*asppc*, pZE-pP_LlacO1_-EP-pP_MA_-*asppc*, and pZE-pP_MA_-EP-pP_MA_-*asppc*, respectively (see Supplementary Table 2 for a list of all asRNAs used in this study).

### Cultivation conditions

For muconic acid biosynthesis, all transformants of BW25113/F′ or its knockout mutants were cultured in 3.5 mL LB medium with appropriate antibiotics at 37 °C with shaking at 290 rpm for 7 h. Then 400 µL cultures were transferred into 125-mL baffled flasks containing 20 mL of M9Y media and cultivated at 30 °C with shaking at 290 rpm for 48 h. When needed, isopropyl β-d-1-thiogalactopyranoside (IPTG) was added to the medium during culture transfer with a final concentration of 0.5 mM. 1 mL cultures were sampled to measure cell optical density at 600 nm (OD_600_) and analyze the products by HPLC every 8 h.

### HPLC analysis

Both the standard and samples were analyzed and quantified by reverse phase HPLC (Dionex Ultimate 3000) equipped with a ZORBAX SB-C18 column and an Ultimate 3000 photodiode array detector. Solvent A was water with 0.1% formic acid, and solvent B was methanol. The column temperature was set to 28 °C. The HPLC program was set at a flow rate of 1 mL min^−1^ with gradient concentrations: 5–50% solvent B for 15 min, 50–5% solvent B for 1 min, and 5% solvent B for additional 4 min. Quantification of muconic acid was based on the peak areas at absorbance of 260 nm.

### Fluorescence assays

To examine the dynamic responses of the hybrid MA biosensors to exogenous MA (see Supplementary Fig. 1), the corresponding *E*. *coli* BW25113/F′ transformants were cultured in 3.5 mL LB medium with appropriate antibiotics at 37 °C with shaking at 290 rpm for 7 h. Then 70 μL cultures were transferred into 3.5 mL of LB media with various concentrations (0 to 10 mM) of muconic acid and appropriate antibiotics at 37 °C with shaking at 290 rpm for 24 h. 40 μL cultures were sampled (at 6, 12, and 24 h) and transferred into a black 96-well plate with clear bottom (BRAND plates) containing 160 μL of diH_2_O for fluorescence assay.

To characterize the P_MA_-CatR system (Fig. 1) and identify the effect of CatR abundance on the P_MA_ dynamic responses (see Supplementary Fig. 2), the corresponding *E. coli* transformants were cultured in 3.5 mL LB medium with appropriate antibiotics at 37 °C with shaking at 290 rpm for 7 h. Then 4 μL cultures were transferred into a black 96-well plate with clear bottom (BRAND plates) containing 200 μL of LB media with various concentrations of muconic acid (0, 0.2, 1.0, 5.0 mM) and appropriate antibiotics. The plate was incubated in the Synergy HT (BioTek) plate reader with medium continuous shaking at 37 °C for 24 h.

For fluorescence assays, the cell optical density was measured at absorbance of 600 nm and fluorescence intensity was recorded using the following parameters. The eGFP fluorescence intensity was detected by using an excitation filter of 485/20 nm and an emission filter of 528/20 nm. The RFP fluorescence intensity was detected by using an excitation filter of 530/25 nm and an emission filter of 590/35 nm. The measurement interval was set to be 40 min for each sample. The BW25113/F′ strains containing the corresponding empty plasmids were used as blank groups to measure background fluorescence. The reading type was set as endpoint mode. The reference signal gains were set as 2000 when characterizing upregulation or downregulation only in Fig. 1c or d, respectively. However, to avoid the fluorescence signal exceeding the machine’s upper limit and provide an optimal window, the reference signal gain was set as 2000 in Fig. 1e and set as 200 in Fig. 1f when characterizing both upregulation and downregulation in an RFP/GFP dual fluorescence reporter system.

### Statistics

All error bars were presented as s.d. of replicates. The number of replicates was reported in the corresponding figure legend. Where relevant, two-tailed *t*-tests were used to determine statistical significance between different groups. A *P*-value < 0.05 was considered to be statistically significant. A very small portion of data (<5%) were excluded because they significantly deviated from the corresponding averages. The transformant colonies used for data collection were randomly selected from the agar plates.

### Data availability

The datasets generated and/or analyzed during the current study are available from the corresponding author on reasonable request.

## Electronic supplementary material

Supplementary Information
