# Supplementary material for: Sensor-regulator and RNAi based bifunctional dynamic control network for engineered microbial synthesis
Source: Nat Commun. 2018 Aug 2;9:3043. doi: 10.1038/s41467-018-05466-0 (PMC6072776; doi:10.1038/s41467-018-05466-0)
Supplement: Supplementary file 1 — Supplementary Information [file 41467_2018_5466_MOESM1_ESM.docx]

**Sensor-regulator and RNAi based bifunctional dynamic control network for engineered microbial synthesis**

Yang *et al.*

**
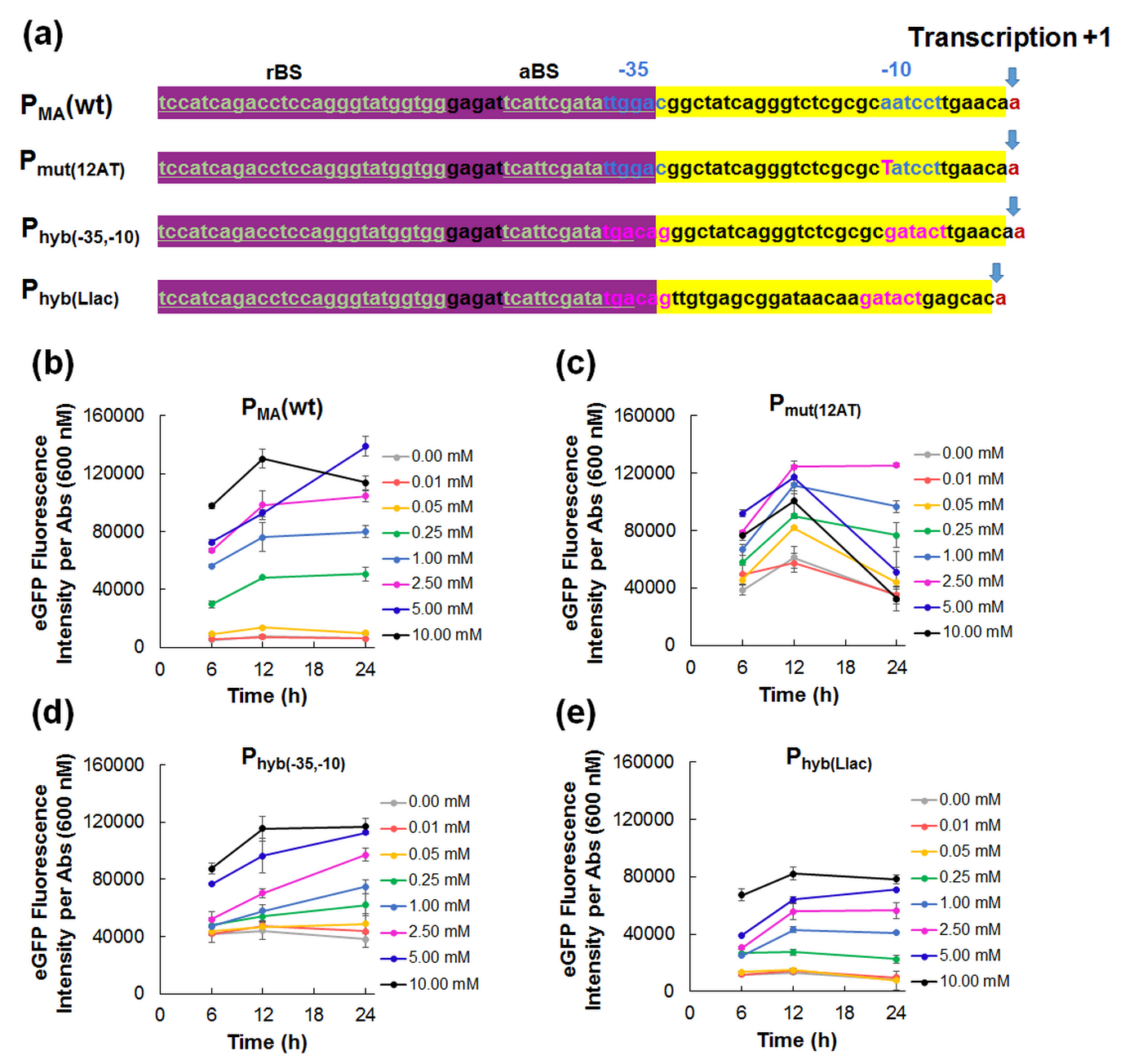
**

**Supplementary Figure 1.** Development of hybrid promoters responsive to muconic acid (MA). (a) Design of the hybrid P_MA_ promoters. The DNA sequences in the purple boxes contain the CatR repression binding site (rBS) and activation binding site (aBS). The sequences in blue indicate -35 and -10 regions of the wild type P_MA_ promoter. Transcription start sites are colored by pink and pointed by blue arrows. P_mut(12AT)_ represents a mutated P_MA_ promoter and is the wild type P_MA_ promoter with a mutation (A to T) at -12 position. P_hyb(-35,-10)_ represents a hybrid P_MA_ promoter and is the wild type P_MA_ promoter with -35 and -10 regions replaced by those of P_LlacO1._ P_hyb(Llac)_ also represents a hybrid P_MA_ promoter and is the wild type P_MA_ promoter with the sequence spanning from -35 region to the transcription start point completely replaced by that of P_LlacO1_. (b-e) Dynamic responses of the developed MA biosensors to exogenous MA. All the above data were generated from three biological replicates, and error bars represent standard deviations. Gray: 0 mM MA, Coral: 0.01 mM, Yellow: 0.05 mM, Green: 0.25 mM, Blue: 1.00 mM, Pink: 2.50 mM, Purple: 5.00 mM, Black: 10.00 mM.


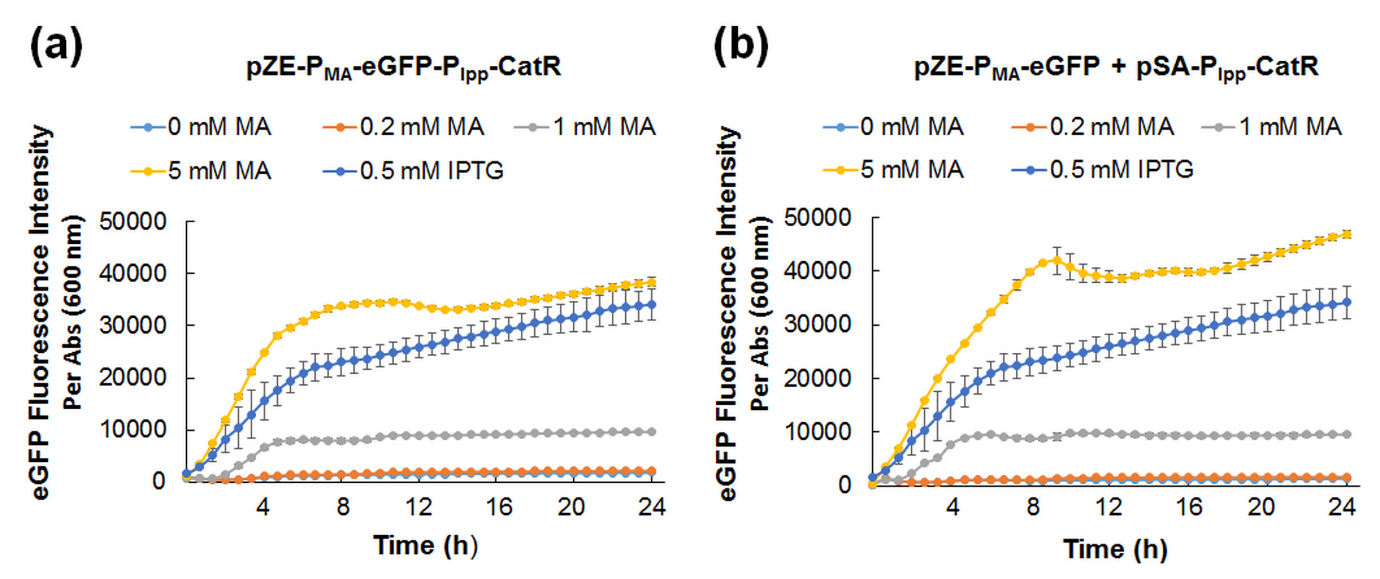


**Supplementary Figure 2.** The impact of CatR abundance on the P_MA_ dynamic response. (a) The P_MA_ dynamic response with a high concentration of CatR. (b) The P_MA_ dynamic response with a low concentration of CatR. All the above data were generated from four biological replicates, and error bars represent standard deviations.

**Supplementary Table 1.** Strains and plasmids used in this study

| **Strain** | **Genotype** | **Source** |
| --- | --- | --- |
| *E. coli* XL1-Blue | *recA1 endA1 gyrA96 thi-1 hsdR17 supE44 relA1 lac*[*F'proAB lacI^q^ZΔM15 Tn10 (Tet^r^)*] | Stratagene |
| *E. coli* BW25113/F' | *rrnBT14* Δ*lacZWJ16 hsdR514* Δ*araBADAH33* Δ*rhaBADLD78* | 1 |
| GA0 | *E. coli* BW25113/F' integrated with P_LlacO1_-eGFP cassette between *nupG* and *speC* loci | 2 |
| YYP167 | *E. coli* BW25113/F' with *pykF* and *pykA* disrupted | This study |
| YYP171 | YYP167 with *ppc* disrupted | This study |
| **Plasmid** | **Description** | **Source** |
| pZE12-luc | P_LlacO1_, colE ori, luc, Amp^r^ | 3 |
| pCS27 | P_LlacO1_, P15A ori, Kan^r^ | 3 |
| pSA74 | P_LlacO1_, pSC101 ori, Cm^r^ | 3 |
| pZE-pP_lpp_-CatR | pZE12 harboring CatR under the control of P_lpp_ promoter | This study |
| pZE-pP_LlacO1_-eGFP | pZE12-luc harboring P_LlacO1_-eGFP | 4 |
| pZE-pP_MA_-eGFP | pZE12-luc harboring P_MA_-eGFP | This study |
| pZE-pP_MA_-eGFP-pP_lpp_-CatR | pZE12-luc harboring P_MA_-eGFP and P_lpp_-CatR | This study |
| pZE-pP_mut(12AT)_-eGFP-pP_lpp_-CatR | pZE12-luc harboring P_mut(12AT)_-eGFP and P_lpp_-CatR | This study |
| pZE-pP_hyb(-35,-10)_-eGFP-pP_lpp_-CatR | pZE12-luc harboring P_hyb(-35,-10)_-eGFP and P_lpp_-CatR | This study |
| pZE-pP_hyb(Llac)_-eGFP-pP_lpp_-CatR | pZE12-luc harboring P_hyb(Llac)_-eGFP and P_lpp_-CatR | This study |
| pSA-pP_lpp_-CatR | pSA74 harboring CatR under the control of P_lpp_ promoter | This study |
| pZE-pP_LlacO1_-PT | pZE12-luc harboring PT sequence under the control of P_LlacO1_ promoter | 4 |
| pZE-pP_MA_-PT | pZE12-luc harboring PT sequence under the control of P_MA_ promoter | This study |
| pZE-pP_MA_-as*egfp* | pZE-pP_MA_-PT harboring 100 bp as*egfp* DNA under the control of P_MA_ promoter | This study |
| pCS-pP_MA_-RFP | pCS27 harboring P_MA_-RFP | This study |
| pZE-pP_LlacO1_-EP | pZE12-luc harboring *entC* and *pchB* under the control of P_LlacO1_ promoter | 3 |
| pSA-pP_LlacO1_-NC | pSA74 harboring *nahG^opt^* and *catA* under the control of P_LlacO1_ promoter | 3 |
| pZE-pP_MA_-EP | pZE12-luc harboring P_MA_-EP | This study |
| pSA-pP_LlacO1_-NC-pP_lpp_-CatR | pSA74 harboring P_LlacO1_-NC and P_lpp_-CatR | This study |
| pZE-pP_LlacO1_-EP-pP_MA_-as*pykF*-pP_MA_-as*pykA* | pZE12-luc harboring P_LlacO1_-EP, P_MA_-as*pykF* and P_MA_-as*pykA* | This study |
| pZE-pP_LlacO1_-EP-pP_MA_-as*ppc* | pZE12-luc harboring P_LlacO1_-EP, P_MA_-as*ppc* | This study |
| pZE-pP_LlacO1_-EP-pP_LlacO1_-as*ppc* | pZE12-luc harboring P_LlacO1_-EP, P_LlacO1_-as*ppc* | This study |
| pZE-pP_MA_-EP-pP_MA_-as*ppc* | pZE12-luc harboring P_MA_-EP, P_MA_-as*ppc* | This study |

**Supplementary Table 2.** asRNA sequences used in this study.

| **Sequence** | **Description** | | | **Source** | |  |
| --- | --- | --- | --- | --- | --- | --- |
| gaattcAGGAGGAATTAACCATGCAGTGGTGGTGGTGGTGGTGGGTACCGATATCAAGCTTGCGGCCGCCATGGCATGCGATATCAAGCTTGCGGCCGCCATGGATCCCACCACCACCACCACCACTGCATGGTTAATTCCTCCTtctaga |  |  | ApoI-PT1-Acc651 4  -MCI-BamHI-PT2  -XbaI | | | |
| ggtaccCATTAACATCACCATCTAATTCAACAAGAATTGGAACAACTCCAGTGAAAAGTTCTTCTCCTTTACTCATGGTACCTTTCTCCTCTTTAATGAATTCGGTggatcc |  |  | Acc651-as*egfp*  (100 bp)-BamHI | | 4 | |
| ggtaccGCATTTTAGCTAACATCTCTTCAGATTCGGTTTTCGGTCCGATGGTGCAAACAATTTTGGTCTTTTTCATGACAGTCTTAGTCTTTAAGTTGAGAAGGATggatcc |  |  | Acc651-as*pykF*  (100 bp)-BamHI | | This Study | |
| ggtaccGATTATTATCGCGATCTGTTGCTGGGCCTAACGTGGTAACGATTTTTGTTCTGCGAAGCCTTCTGGACATGTAATACTCCGTTGACTGAAACAACCAGGTggatcc |  |  | Acc651-as*pykA*  (100 bp)-BamHI | | This Study | |
| ggtaccTGATGGTTTCTCCCAGCACTTTGCCGAGCATACTGACATTACTACGCAATGCGGAATATTGTTCGTTCATATTACCCCAGACACCCCATCTTATCGTTTGggatcc |  |  | Acc651-as*ppc*  (100 bp)-BamHI | | This Study | |

**Supplementary References**

1. Atsumi, S., Cann, A. F., Connor, M. R., Shen, C. R., Smith, K. M., Brynildsen, M. P., Chou, K. J., Hanai, T., Liao, J. C.. Metabolic engineering of *Escherichia coli* for 1-butanol production. *Metab Eng* **10**, 305-311 (2008).
2. Wang, J., Wu, Y., Sun, X., Yuan, Q. & Yan, Y. De Novo Biosynthesis of Glutarate via alpha-Keto Acid Carbon Chain Extension and Decarboxylation Pathway in *Escherichia coli*. *ACS Synth. Biol.* **6**, 1922-1930, (2017).
3. Lin, Y.H., Sun, X.X., Yuan, Q.P. & Yan, Y.J. Extending shikimate pathway for the production of muconic acid and its precursor salicylic acid in *Escherichia coli*. *Metab Eng* **23**, 62-69 (2014).
4. Yang, Y.P., Lin, Y.H., Li, L.Y., Linhardt, R.J. & Yan, Y.J. Regulating malonyl-CoA

metabolism via synthetic antisense RNAs for enhanced biosynthesis of natural products. *Metab Eng* **29**, 217-226 (2015).
